# The Cautious Promise of GLP-1 Receptor Agonists

**DOI:** 10.31486/toj.24.5045

**Published:** 2024

**Authors:** Rachel S. Dauterive

**Affiliations:** Department of Bariatric Surgery, Ochsner Clinic Foundation, New Orleans, LA

At this point, it is difficult to find someone who hasn’t heard of semaglutide or tirzepatide. Most people know a friend or family member who is taking one of these medications, or they have taken one of these drugs themselves. These drug names are all over social media. They are all over mainstream media. A quick Google search returns countless sites offering prescriptions for compounded semaglutide or tirzepatide mailed right to your home.

The US Food and Drug Administration (FDA) originally approved glucagon-like peptide-1 (GLP-1) receptor agonists for the treatment of type 2 diabetes in 2005, and since then, the potential for additional applications has become evident as this class of medications has evolved. In 2014, Saxenda (liraglutide), a daily injection, became the first GLP-1 receptor agonist to be approved for the treatment of obesity. Ozempic (semaglutide), a weekly injection, was approved for type 2 diabetes in 2016. Even though Saxenda had already been approved for obesity treatment, the use of GLP-1 receptor agonists for weight loss really picked up steam with Ozempic, perhaps because Saxenda was a daily injection, while Ozempic was a weekly injection. Or perhaps Saxenda just did not get the media coverage that Ozempic received. But because of the popularity of semaglutide among celebrities and the accompanying media coverage, the drug's use for weight loss exploded.

Following the trial STEP 1: Research Study Investigating How Well Semaglutide Works in People Suffering From Overweight or Obesity,^1^ the FDA approved semaglutide for the treatment of obesity in 2021, and the drug was marketed as Wegovy. The STEP 1 study demonstrated an average body weight loss of 15% with the use of Wegovy 2.4 mg weekly. When Wegovy first became available, many patients were able to purchase it at a discounted price with a manufacturer coupon. But once the coupon expired, at a cost of approximately $1,600 per month and coverage provided by few insurance plans, Wegovy simply was not an option for most patients.

When I first started practicing obesity medicine around 2020, I frequently prescribed Ozempic off-label for weight loss. At that time, the supply of Ozempic was not limited as it is today, and the drug was commonly covered by insurance plans without prior authorization. Although Wegovy became available in 2021, it was cost prohibitive for most patients, so Ozempic continued to be prescribed off-label for weight loss. However, as the popularity of Ozempic grew, there started to be supply shortages. Pharmacies were unable to keep Ozempic in stock, which became a problem for patients using it for the treatment of type 2 diabetes. In addition, insurance companies began requiring prior authorization for Ozempic and only approved the medication for patients with type 2 diabetes. As a result, many patients who were taking the medication for weight loss were no longer able to obtain Ozempic, and without a medication of similar effectiveness and affordability to replace it, many of these patients started to regain weight.

Meanwhile, the GLP-1 receptor agonists were evolving to dual GLP-1 plus glucose-dependent insulinotropic polypeptide (GIP) receptor agonists with the medication tirzepatide. Following a similar trajectory as semaglutide, tirzepatide was first approved by the FDA for the treatment of type 2 diabetes in May 2022 as Mounjaro. Then the SURMOUNT-1 study^2^ demonstrated an average body weight loss of approximately 20% with a 15 mg weekly injection of tirzepatide, leading to the drug's approval as Zepbound for the treatment of obesity. As with Ozempic and Wegovy, there are intermittent supply shortages of Zepbound. Insurance coverage is also lacking, making Zepbound cost prohibitive for many patients.

I am a big proponent of preventive medicine and lifestyle medicine. As physicians know, most chronic diseases are attributable to lifestyle habits—lack of activity, poor dietary habits, tobacco and alcohol use/abuse, and lack of sleep. More than 40% of the US population is obese, and with obesity comes increased risk of cardiovascular disease, type 2 diabetes, liver disease, musculoskeletal disease, sleep apnea, and many cancers. For many patients who have tried and failed various diets and weight loss programs, the GLP-1 receptor agonists have helped them meet their weight loss goals and improve their overall health. These medications enable patients to more easily and consistently reduce their food intake and thereby lose weight. I have seen patients who were able to stop taking medications for hypertension and patients who reduced their insulin requirements, normalized their A1c levels, reversed fatty liver disease, and reduced inflammation. In fact, Wegovy is now approved for its cardioprotective benefit in patients with a history of cardiovascular disease (specifically those having had a stroke, myocardial infarction, or peripheral artery disease). Semaglutide is even being studied as a possible treatment for dementia. A 2024 study showed a reduction in sleep apnea severity in patients using tirzepatide.^3^ It certainly seems that these medications have the potential for far-reaching health benefits.

One of the most interesting effects of these medications is not only the reduction in appetite and cravings, but for most people the *food noise*—the almost obsessive and constant thinking about food—is quieted. One of my patients reported that he is more productive at work because he is not thinking about food all the time. In addition, many patients have reported a lack of interest or desire for alcohol. They no longer need or want a drink at the end of the day or when out to dinner with friends. And it is not just a desire for food and beverages that is reduced. I’ve even had a patient report that she is shopping less on Amazon.

Of course, as with any medication, there are adverse side effects, although the GLP-1 receptor agonists are generally well tolerated. Gastrointestinal side effects are the most common. Many patients are concerned about “gastric paralysis.” One of the ways these medications work is by delaying gastric emptying, thereby causing a feeling of fullness with smaller portions. However, use of the word *paralysis* insinuates a more extreme situation than what actually occurs. Other common side effects include nausea and vomiting. Many female patients liken this side effect to morning sickness when pregnant. GLP-1 receptor agonist side effects can be controlled for the most part by eating small portions; avoiding high-fat, greasy foods; and eating at regular intervals. Constipation is another commonly reported side effect. For most patients, adequate fluid and fiber intake can prevent constipation. In fact, eating a healthy diet and staying properly hydrated can reduce the likelihood of experiencing many of the commonly reported adverse side effects.

One of the most commonly expressed concerns by patients using GLP-1 receptor agonists for weight loss is whether the weight loss can be maintained when the medication is discontinued. It has become clear that weight regain occurs when patients stop taking the medication. This effect was shown in the STEP 4 trial^4^ in which all participants received semaglutide for the first 20 weeks and then were randomized to either a placebo group or a semaglutide group. Those in the placebo group had an average body weight increase of 6.9%. I also see this effect clinically. Patients frequently report increased appetite once they stop taking the medication, resulting in increased food intake and weight gain. However, I am also concerned about weight regain even when the medication is continued long term. Will patients develop a tolerance to the appetite-suppressing effects after taking one of these medications for 5 or more years? I suppose time will tell.

Obesity is a chronic disease and should be treated as such. While these medications certainly have the potential to provide life-changing benefits for many people, there is still so much that we don’t know about them, including any long-term effects of these drugs. As such, I am hesitant to label the GLP-1 receptor agonists with the term “miracle drug,” at least not yet. Nevertheless, I have seen the positive impact that taking these medications has had on a lot of people in helping them change their eating habits, lose weight, and improve their health. So I continue to recommend and prescribe these medications to my patients who are struggling with obesity. Keep in mind that these medications are truly in their infancy, and it will be interesting to follow the future development and applications for this medication class.
